# Eco-Friendly Sustainable Fluorescent Carbon Dots for the Adsorption of Heavy Metal Ions in Aqueous Environment

**DOI:** 10.3390/nano10020315

**Published:** 2020-02-12

**Authors:** Musa Yahaya Pudza, Zurina Zainal Abidin, Suraya Abdul Rashid, Faizah Md Yasin, A. S. M. Noor, Mohammed A. Issa

**Affiliations:** 1 Department of Chemical and Environmental Engineering, Faculty of Engineering, Universiti Putra Malaysia, Serdang 43400, Selangor, Malaysia; suraya_ar@upm.edu.my (S.A.R.); fmy@upm.edu.my (F.M.Y.); academicfinest@gmail.com (M.A.I.); 2 Department of Computer and Communication System Engineering, Faculty of Engineering, Universiti Putra Malaysia (UPM), Serdang 43400, Selangor, Malaysia; ashukri@upm.edu.my

**Keywords:** carbon dots, heavy metals, adsorption, characterization, environment, sustainability

## Abstract

The materials and substances required for sustainable water treatment by adsorption technique, are still being researched widely by distinguished classes of researchers. Thus, the need to synthesize substances that can effectively clean up pollutants from the environment cannot be overemphasized. So far, materials in bulk forms that are rich in carbon, such as biochar and varieties of activated carbon have been used for various adsorptive purposes. The use of bulk materials for such purposes are not efficient due to minimal surface areas available for adsorption. This study explores the adsorption task at nano dimension using carbon dots (CDs) from tapioca. The properties of carbon structure and its influence on the adsorptive efficacy of carbon nanoparticles were investigated by energy-dispersive spectroscopy (EDS), X-ray photoelectron spectroscopy (XPS), Fourier transform infrared spectroscopy (FTIR), high resolution transmission electron microscopy (HrTEM), and atomic force microscopy (AFM). The results implied carbon present in CDs are good adsorbents for effective adsorption of heavy metal ions (lead) with removal efficiency of 80.6% in aqueous environment. The adsorption process as explored by both Langmuir and Freundlich isotherms have proven favorability of the adsorption process. Langmuir form two and three have correlation coefficients R^2^ at 0.9922 and 0.9912, respectively. The Freundlich isotherm confirms CDs as having defined surface heterogeneity and the exponential distribution of active sites. The adsorption of lead unto CDs obeyed the second order kinetic model with coefficient of determination, R^2^ of 0.9668 and 0.9996 at an initial lead concentration of 20 mg/L and 100 mg/L, respectively. The findings validated the efficiency of CDs derived from tapioca as an excellent material for further utilization in the environmental fields of wastewater pollution detection and clean up, bio-imaging, and chemical sensing applications.

## 1. Introduction

Carbon dots (CDs) are dimensionless nanoparticles classified as carbon nanomaterials with >10 nm in size and are considered latest class of fluorescent nanoparticles [1,2,3]. CDs derived from green and sustainable materials are essential aspects of biology, chemistry, and physical sciences with essential applications in computer science and electronic [4,5,6,7]. CDs have several characteristics such as being environmentally friendly, easy to synthesize, non-blinking, high biocompatibility, durability, high photostability, quenchable (on/off) emission with excitation wavelength that can be functionalized based on their desired applications, and dissolve easily in water with a high carbon content (up to 99.9%) [8]. These attributes of CDs have made it an interesting substance to a wide range of researchers [9,10,11].

Comparatively, semiconductor nano crystals otherwise known as quantum dots (QDs) are nanoparticles with diameter ranging from 1–10 nm. When compared to CDs, QDs are toxic and expensive [12]. QDs are conventional fluorescent dyes that have unique optical properties, however, CDs are the best option due to their high degree of biocompatibility, cost effectiveness, non-toxic, and have been successfully applied in bio imaging, bio sensing [13,14,15,16], agricultural diagnosis sectors [17], pharmaceuticals [18,19], solar cells [20], electrochemical functions [21,22,23], wastewater treatment [24], photocatalysis, and chemical sensing [25,26].

There are numerous techniques for synthesizing CDs, such as; arc-discharge [9,21], laser ablation [8,27,28], chemical oxidation [16,29,30,31], and electrochemical [32,33,34,35,36]. However, several factors need to be considered when adopting a synthesis route to obtain CDs [37]. In the synthesis process of CDs there is the possibility of carbonaceous aggregation that tends to form during carbonization process. This challenge is remedied by synthesis processes such as; hydrothermal route [38,39,40,41], organic pyrolysis, and microwave assisted method [42,43,44,45,46]. These techniques possess the abilities to control the size and uniformity of CDs in solvents [37].

Literature provides reports on the applications of agro-based wastes to synthesize CDs such as; cooking oil waste [38], pomelo peal [45], egg-white and egg-yolk [40], orange juice [37], as well as eggshells [44]. Contrary to ethics of reuse of waste material, these biomass, creates residual waste that are toxic. Hence, the need for clean materials in the synthesis of CDs have became a necessity in the modern era of nanoscale materials. It is advantageous to adopt the use of clean materials in the synthesis of CDs for applications such as bioimaging, drug delievry, water treatment, and for other numerous portable uses. It is not ethical to adopt waste materials/biomass for purposes that relates to health and the environment by process that further releases toxins. The noticeable scientific shortfall of applying biomass in the synthesis of CDs is their lack of essential purity and structural homogeneity to obtain homogenous CDs. Hence, it is necessary to apply clean food materials in the synthesis of homogeneous fluorescent CDs [12]. 

Alqadami et al. [46] applied trisodium citrate based magnetite nanocomposite at pH 4 for the effective removal of toxic dye from aquoues medium in 40 min. Adsorptive performance of Fe_3_O_4_@AMCA-MIL-53 (Al) nanocomposite was utilized for the adsorptive removal of highly toxic methylene blue (MB) and malachite green (MG) dyes from aqueous environment [47]. Xiangtao et al. [48] reported the use of nano materials in batch adsorption process of lead ions. The effective use of nanomaterials have been used widely in detection of pollutants [24] and fluorescence quenching [18], chiefly by electrochemical methods of diffusion layer [24] which technically occurs by the adsorption mechanism [11,21,31,45]. In the present study, tapioca was utilized in the synthesis of homogeneous water soluble fluorescent CDs through an improved hydrothermal method. Characterization analysis of the compositional elements, dimensional sizes, and functional groups were analysed for adequate understanding of the structural mechanism of CDs for further application in the adsorption of heavy metal ions (lead).

## 2. Materials and Methods 

Acetone along with sodium hydroxide were of analytical grade obtained from Sigma-Aldrich. (Saint Louis, MO, USA) Tapioca was locally sourced. A 50 mL stainless steel capacity of hydrothermal reactor was used. Convective oven, 365 nm Uv-Lamp, Centrifuge machine, lead (ll) nitrate (Pb(NO_3_)_2_) analytical reagent, and M.W. 331.21, were obtained from R & M marketing, Essex, U.K. Solutions used in this study were prepared with deionized water (DI) purified by a Milli-Q water type system (18 MΩ cm).

### 2.1. Synthesis of Carbon Dots

**Figure 1:**
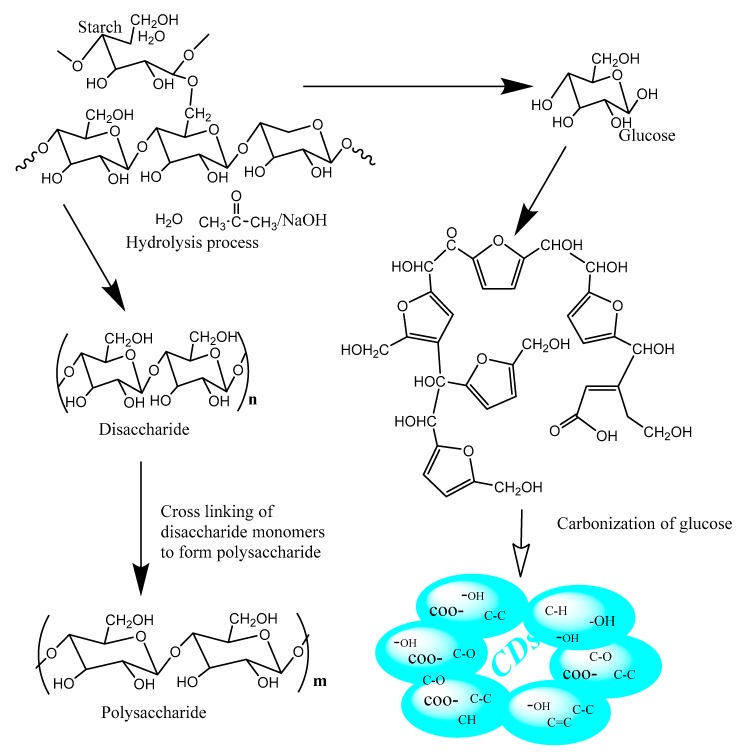
Carbon dots synthesis mechanism.

Tapioca flour (0.1 g) was mixed with 16mL of solvent (D.I H_2_0**/**NaOH**/**(CH_3_)_2_CO). The mixture was oven heated at 175 °C in a convective oven for 1.45 h which yielded disaccharide and glucose (Figure 1).

The disaccharide crosslinked with several other disaccharides to form polysaccharides (subsequently removed as residue by centrifugation at 3000 rpm for 20 min), while the glucose went through carbonization that produced the needed CDs with functional groups (COO–, C=O, –OH, C–H, C=C, C–O–C) afloat on the surfaces of the synthesized CDs. The mechanism of the synthesized CDs is as displayed in Figure 1.

### 2.2. Carbon Dots Characterization

The characterization of CDs by Fourier-transform Infrared spectroscopy (FTIR) was conducted by Perkin Elmer spectrum 100 spectrometer (Bridgeport avenue Shelton, CT, USA). The measurement is made by attenuated total reflection method with frequency range of 4000–500 cm^−1^, that allows for the direct measurement of functional groups in CDs. The absorption spectra were taken from a UV-spectrometer 1800 series. High resolution transmission electron microscopic (HrTem) and energy-dispersive spectroscopy (EDS) were characterized by FEI Tecnai G2 F20, 200 kV, ZrO2/W (100) z-contrast dark-field stem imaging by the HAADF detector. The zeta potential analysis was carried out using zetasizer Nano series (Malvern). Finally, X-ray photoelectron spectroscopy (XPS) was conducted with ULVAC-PHI Quantera II and the X-ray source is Al K-Alpha, monochromatic source (1486.6 eV).

### 2.3. Adsorption Studies

Different concentrations of lead (ll) nitrate (Pb(NO_3_)_2_) at 20 mg/L, 40 mg/L, 60 mg/L, 80 mg/L, and 100 mg/L were prepared with deionized water. These various concentrations of Pb(NO_3_)_2_ solution were mixed with CDs at room temperature (25 °C). The solution was next centrifuged at 120 rpm and the supernatant was taken for measurement using UV Spectrometer. The application of CDs for adsorption of lead was conducted by batch adsorption process. The initial concentrations of lead and the amount absorbed by CDs have been reported in the later sections of the article. The mass of CDs is the mass of the tapioca used for the synthesis of CDs (0.1 g) while the contact time and pH were varied from 5–260 min and 3–12, respectively. The amount adsorbed at equilibrium, *q_e_* (mg/g) and the percentage removal of lead were calculated as in literature [46,47].

(1) $$q_{e}=\frac{\left(c_{0}-c_{f}\right)v}{M}$$

where, *q_e_* is equilibrium concentration of lead at any time (mg/g), *M* is mass of the adsorbent used (g), and *v* is volume of the lead solution (L)

(2) %Removal=c0−cfc0×100

where, *C*_0_ is initial lead concentration in sample (mg/L), *C_f_* is equilibrium lead concentration in final solution after adsorption (mg/L).

### 2.4. Adsorption Isotherm

Adsorption isotherm describes the equilibrium relationship between adsorbent (CDs) and the adsorbate (lead). It indicates the amount of lead (Pb^2+^) adsorbed by the CDs and the residual lead in the bulk solution at room temperature [49]. The equilibrium isotherms were obtained graphically by plotting solid phase concentration against liquid phase concentration [50].

#### 2.4.1. Langmuir Isotherm, Adsorption Equation, and Limitations

Langmuir isotherm is one of the famous studies applied for adsorption capacities [49]. It assumed that adsorption is monolayer and reversible. The adsorption is homogenous when it takes place within specific site while the energy is constant and does not depend on the degree of occupation of adsorbents at active center [51]. All adsorption sites are equivalent with no interactions between adsorbate molecules on adjacent sites.

The following equation is Langmuir isotherm Equation [50].

(3) $$q_{e}=\frac{q_{max}K_{L}C_{e}}{1+C_{e}K_{L}}$$

where:

*q_e_* = adsorption capacity (mg/g)
*q_max_* = maximum monolayer adsorption capacity of the adsorbent (mg/g)
*C_e_* = equilibrium concentration of the adsorbate (mg/L)
*K_L_* = Langmuir adsorption constant related to free energy adsorption (L/mg)

Since the estimation of the adsorption isotherm parameters interference are through linearization method. The following three forms of linearized isotherm equation are used to determine the constants *K_L_* and *q_max_* [52].

Form 1:

(4) $$\frac{C_{e}}{q_{e}}=\frac{1}{q_{max}K_{L}}+\frac{C_{e}}{q_{max}}$$

Form 2:

(5) $$\frac{1}{q_{e}}=\frac{1}{q_{max}}+\frac{1}{q_{max}K_{L}C_{e}}$$

Form 3:

(6) $$q_{e}=-\frac{q_{e}}{C_{e}K_{L}}+q_{max}$$

Equations (4)–(6) are the derivation of Langmuir isotherm model. It clarifies what happens at the equilibrium of monolayer adsorption process. The number of molecules being adsorbed will be equal to the number of molecules leaving the adsorbed site, that is total amount absorbed *q_max_* is directly proportional to concentration in solution and available area for adsorption. The constants can be evaluated from intercept and the slope of linear plots of experimental data of (*C_e_*/*q_e_*) versus *C_e_* (for Equation (4)) or (1/*q_e_*) versus (1/*C_e_*) (for Equation (5)) or *q_e_* versus (*q_e_*/*C_e_*) (for Equation (6)). The most popular form of analyzing adsorption equilibrium data is Langmuir Equation (3) [49,50].

#### 2.4.2. Freundlich Isotherm

Freundlich isotherm is an empirical relationship that can be utilized for non-ideal sorption that involves heterogeneous surface energy system [49]. It is assumed that there are neither homogeneous site energies nor limited level of sorption [47]. Freundlich isotherm equation is expressed by the following Equation [49].

q_e_ = K_F_ C_e_^1/n^ (7)

q_e_ = adsorption capacity (mg/g)
K_f_ = Freundlich isotherm constant (mg/g)
C_e_ = equilibrium concentration of adsorbate (mg/L)
^1/n^ = adsorption capacity (L/mg)

By linearizing Equation (8), constants *K_F_* and *n* were determined using ln *q_e_* versus *ln C_e_* plot.

(8) $$ln\;q_{e}=\ln K_{F}+\frac{1}{n}lnC_{e}$$

*K_F_* is the Freundlich proportionality constant obtained from the intercept and 1/*n* from the slope. Here *K_F_* represents the quantity of the absorbed lead required to maintain lead concentration in the solution at unity (i.e., 1 mg/L). As *K_F_* increases, the adsorption capacity for a given adsorbate also increases. The slope 1/*n* ranges between zero and one and indicates intensity of adsorption or surface uniformity. Higher value of 1/*n* (or lower *n*) means adsorption is more favorable and greater surface heterogeneity [46].

## 3. Results

### 3.1. Characterization of Carbon Dots (CDs)

#### 3.1.1. Atomic Force Microscopy (AFM) and High Resolution Transmission Electron Microscopy (HrTem) of Carbon Dots (CDs)

It is essential to determine the particle sizes of the synthesized CDs by utilizing precision techniques such as the atomic force microscopy (AFM) and HrTem [25]. The synthesis of CDs with optimal clearance and abundant surface sites are essential for research applications in adsorption by nano processes [39].

**Figure 2:**
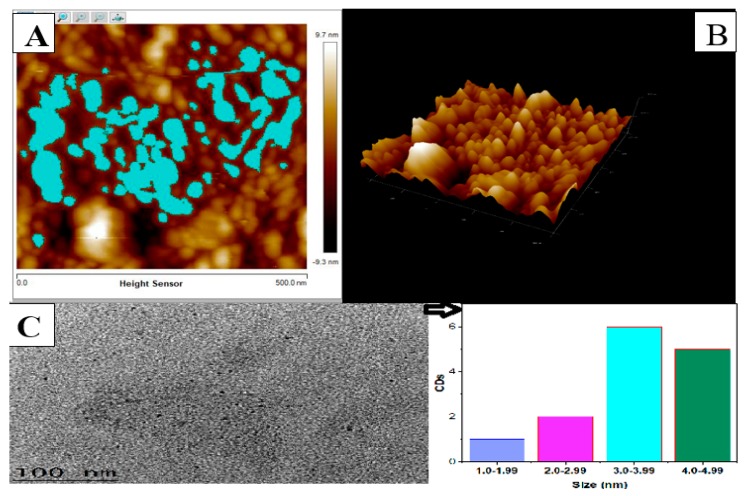
(**A**) atomic force microscopy (AFM) 2D image of carbon dots (CDs) (**B**) AFM 3D morphology of CDs (**C**) high resolution transmission electron microscopic (HrTEM) image of CDs.

Figure 2A,B display 2D and 3D plots of the morphological pattern of carbon dots comprising 32 counts of CDs with mean heights and diameters of 2.440 nm and 32.387 nm, respectively. The AFM images provide the nature of CDs morphological structure. This is important, as it demonstrates the ability of the particles to adsorb heavy metal pollutants that are present in aqueous medium. This is in agreement with previous studies of adsorption isotherms and kinetic models [53].

Figure 2C, is the HrTEM images of CDs with their size distributions. The sizes of CDs on the inset histogram shows an average size of 3.0–3.99 nm as the most abundant available size count. More so, the average size of CDs at 3.0–3.99 nm meaning that there is abundant surface area available for adsorption of lead ions.

Table 1 shows a detailed presentation of size distribution of CDs, obtained by measuring the heights of 32-single carbon dots observed under atomic force microscopy. The mean CDs surface of 1801.610 nm^2^ and mean diameter of 32.387 nm provides plentiful adsorption sites for the effective removal of lead ions from aqueous solution as reported in this study.

#### 3.1.2. Zeta Potential of Carbon Dots

The Zeta (ζ) potential of CDs, known as electrokinetic potential of the synthesized fluorescent CDs was measured by zetasizer to obtain the surface charge. This technique also provides insight on CDs properties such as double layer attributes with several hydrophilic functional groups (hydroxyl, carboxyl, and carbonyl).

Zeta potential measurements are occasionally presented as a useful and efficient method of evaluating CDs ability to adsorb heavy metal ions. The surface charge of the carbon dots at pH = 7 was obtained as −31.7 mV charge. The negative ζ potential is due to dense electron cloud concentrating on the CDs [52]. The value of ζ potential relies upon a short or a long-term stability of CDs particles. CDs with a high zeta potential (negative or positive) are considered stable electrostatically [38], while particles with low zeta potentials tend to coagulate or aggregate over a brief time period. A low ζ potential results in a weak physical stability of CDs. Scientifically, when the ζ of carbon dots is high, it means that the repulsive forces have exceeded the attractive forces, which creates a relatively stable system. Highly dispersed nanoparticles such as CDs have zeta potential values greater than +30 mV or less than −30 mV [54]. Particles at nano-dimension having zeta potential values ranging from −10 to +10mV are considered to be neutral [55]. The zeta potential is very sensitive to the changes happening upon dilution, such as the variations in pH and ionic strength [54]. At alkaline pH greater than pH 7, the zeta potential remains highly negative to reflect existence of stable anions. As the pH decreases below seven (acidic region), the zeta potential normally becomes less negative in values until it becomes zero. This is known as the point zero charge whereby potential changes from negative to positive (or positive to negative). After that the zeta potential becomes more positive in values (as pH becomes more acidic) which reflects the existence of positively charged surrounding of the CDs. Hence at greater pH than seven, a stable negatively charge carbon dots dominate and attract positively charge heavy metals cations (lead (Pb^+2^).

#### 3.1.3. Energy Dispersive Spectroscopy (EDS)

**Figure 3:**
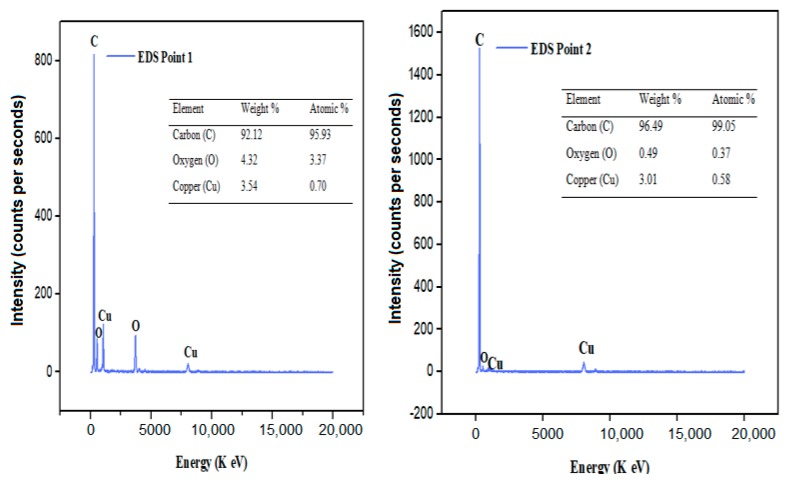
Energy-dispersive spectroscopy (EDS) Spectra of carbon dots at point 1 and 2.

This technique has been applied to obtain the elemental composition of synthesized CDs. The EDS is an integrated feature of HrTEM. The CDs specimen were bombarded with an electron beam within the HrTEM. The bombarding electrons collides with the carbon dots, knocking some of their atoms off in the process, which creates a space, vacated by an ejected inner shell electron. This empty space is eventually occupied by another higher-energy electron from an outer shell. This happens because the transferring outer electron relinquishes some energy by emitting an X-ray. Thus, by measuring the amounts of energy present in the X-rays being released by CDs during electron beam bombardment, the identity of atoms from which the X-ray was emitted were established as shown in Figure 3 [56,57,58,59].

Figure 3 (point one EDs) depicts the analysis of the compositional elements in CDs, the figure shows a high carbon content of 95.93%.

The presence of copper (Cu) is as a result of elemental transfer from the measuring tool to CDs. The Eds peaks from zero to 10 energy level depicts the elements available on CDs where the initial peak is considered the top layer of the EDS that represents carbon (C). The second peak is oxygen (O) while subsequent peaks are the tool piece electrode, made of Copper (Cu) material as earlier stated.

Similarly, Figure 3 (EDS point two) shows the EDs measured on another location point two whereby this time a higher concentration of atomic carbon was recorded than previously discovered on EDS point one. Carbon remained the highest compositional element found in CDs (99.05 atomic %). The abscissa axis of the EDS is considered the ionization energy pathway while the ordinate axis indicates the counts per second of the CDs intensity [55]. The EDS spectrums are plots of X-rays received for each energy level indicated. They display peaks that corresponds to individual levels of energy received as generated by X-rays. Each peak is unique to an atom that corresponds to a single element (C, O, and Cu). A higher peak in the spectrum, denotes the most dominant element present in CDs.

#### 3.1.4. X-ray Photoelectron Spectroscopy (XPS)

The XPS technique is an important concept employed in surface science to provide significant and vital information on the chemical state of elements and relative composition by atomic layers present in CDs. This technique employs electron excitement and electron kinetic energy measurement presented in the form of several peaks and positions in a spectrum pattern. Individual elements are determined by the attributes of the CDs such as spin orbital-splitting and peak area ratio.

**Figure 4:**
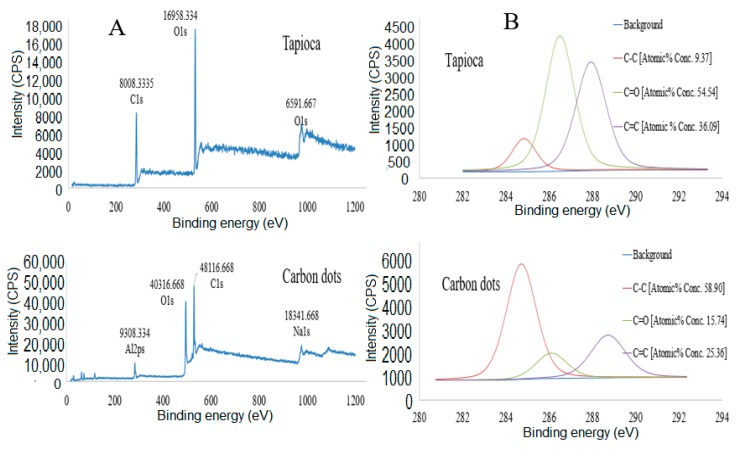
(**A**,**B**) Wide and narrow scan for tapioca (precursor) and synthesized carbon dots.

The XPS results (Figure 4) show clear dominance of carbon at 99.05 atomic percentage (%).

Figure 4A,B shows wide and narrow scan spectrums of tapioca (precursor) and CDs. The XPS wide spectrum (Figure 4A) of CDs at 16,958.334 counts per seconds reflects 57.32% atomic% concentrations of carbon and 8008.3335 counts per seconds of oxygen peaks at 42.68% atomic% concentrations at high photoelectron energies.

There is a significant elemental increase of carbon from 57.32% in tapioca (precursor) to 70.652% in the content of CDs. Furthermore, there is a significant decreased content of oxygen from 42.68% to 21.349% due to the decomposition and intermolecular formation of hydrocarbon rings (cyclization) by hydrothermal reaction [57].

Figure 4B shows a high-resolution of narrow scan for C1s spectra of tapioca (as CDs precursor material) and synthesized CDs. The tapioca validates the presence of C–C, C–O, and C=C bond with atomic concentration of 9.37%, 54.54%, and 36.09%, respectively. Tapioca binding energy (eV) and intensity (c.p.s) for C–C, C–O, and C=C are at 285.018 eV/106.685 c.p.s, 286.618 eV/ 3945.017 c.p.s, and 288.118 eV/ 3151.267 c.p.s, respectively (Figure 4B (Tapioca)). The narrow scan high resolution on C1s spectra (Figure 4B (carbon dots)) shows some significant shifts on the atomic percentages and intensities of C–C, C–O, and C=C. The major peak at 284.755 eV/5167.234 c.p.s, 286.355 eV/1722.329 c.p.s, and 288.955 eV/2378.359 c.p.s correspond to C–C, C–O, and C=C, respectively. While, the atomic percentages of functional groups such as C–C, C–O, and C=C were 58.90%, 15.74%, and 25.36%, respectively [60].

Based on the XPS and EDS elemental analysis, the hybridization and coefficients that exists between the functional groups and carbon core contributed to the luminescent and adsorptive behavior of CDs [61]. In the fields of environmental science and medical application, tapioca-derived CDs that constituted of organic carbon is intrinsically non-toxic [62,63,64] in comparison to quantum dots and hence provides a solution to toxicity concerns.

#### 3.1.5. Fourier Transform Infrared (FTIR) Analysis

**Figure 5:**
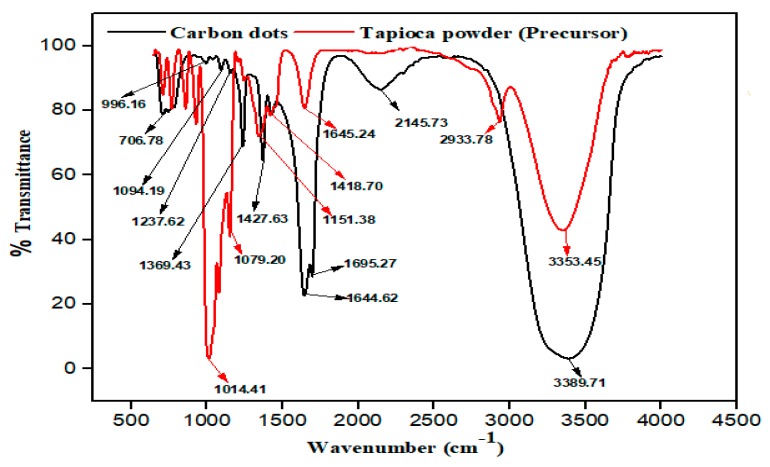
Fourier-transform infrared spectroscopy (FT-IR) spectrum of CDs and tapioca.

Fourier-transform infrared spectroscopy, have been employed in this study to portray and examine the functional structure of CDs. Much more, to reveal the useful compounds of elements present, before and after hydrothermal treatment of the precursor carbon material (tapioca). Figure 5 shows the FTIR spectra of tapioca and carbon dots.

As shown in Figure 5 (tapioca powder), peaks associated with the stretching vibrations of hydroxyl (–OH) and carboxylic (COO–) groups are recorded at 3353.45 and 2933.78 cm^−1^. Further stretching vibration of C-H occurred from 1645.24 to 1418.70. The peaks at 1151.38, 1079.20, and 1014.41 cm^−1^ can be due to the C–H stretching vibrations and out-of-plane bending modes of sp^2^ and sp^3^ –CH group [45].

Figure 5 (Carbon dots), the spectra of Carbon dots, shows the existence of hydroxyl (–OH) group at 3389.71 cm^−1^ which increased on the carbon dots structure as a result of hydrolysis phenomenon. The carboxylic (COO–) group 2145.73 cm^−1^ meanwhile was reduced by thermal destruction of saccharides structure [19,39]. The peaks at 1695.27 cm^−1^ and 1644.62 cm^−1^ indicate the increase in the C–H stretching vibrations of the bending modes of the sp^2^ and sp^3^ –CH group. The peaks around 1427.63 cm^−1^ until 1369.43 cm^−1^ are due to C–O–C [41]. The peak at 1237.62cm^−1^ corresponds to the C=C stretching vibration while 1094.19 cm^−1^ and 996.19 cm^−1^ represents the C=O stretching vibration and the last group at 706.78 cm^−1^ denotes the C=C bond of the unsaturated glucose structure. The FT-IR graph, shows the formation of unsaturated carbon. Along with oxygen-rich groups such as hydroxyl, carboxyl, and carbonyl situated on carbon dots surface, which are in consonance with the hydrothermal process of synthesizing carbon dots [38,39]. The functional groups here identified are responsible for the water-soluble nature of carbon dots [12].

### 3.2. Optical Properties of Carbon Dots (CDs)

**Figure 6:**
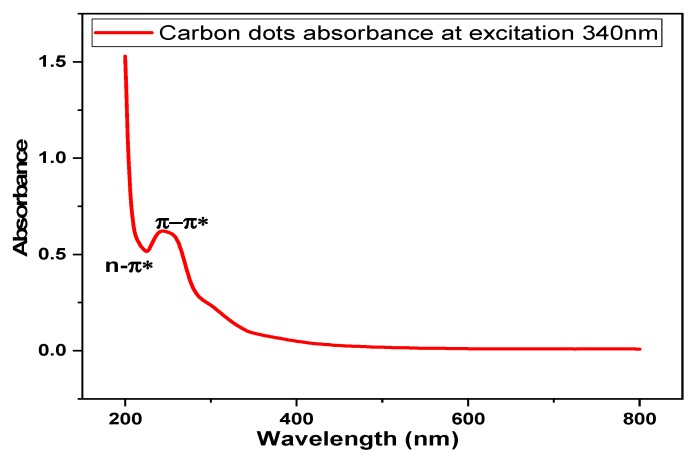
Optical properties of CDs by UV-visible absorption and emission spectra.

The UV-vis spectrometry is a necessary instrument used for validating the quality of CDs [13]. Irradiations of UV-spectroscope on CDs excited by absorbing the energy generated, creates an electron excited state on CDs. The molecules of CDs with extended π-electron provides the basics for the fluorescence emission of CDs (Figure 6). The tapioca-derived CDs is a wavelength dependent photoluminescent ionic solution in the visible range with a surface abundant hydroxyl and carboxylic/carboxyl moieties [13]. The synthesized CDs shows a strong optical absorption in the UV region (230–340 nm) with a tail extending to the visible range (800 nm) as presented in Figure 6.

Absorption shoulders in the spectrum may be due to the π-π*of C=C bonds or n-π* of C=O [37]. The uniqueness of CDs is a result of the fluorescence emitted by it. Based on past study, the dependency of intensity and wavelength emission towards excitation wavelength [15]. This is due to the different size of particles and surface chemistry at different emissive traps of CDs surface which is related to the synthesis method.

### 3.3. Adsorption of Lead Using Carbon Dots (CDs)

**Figure 7:**
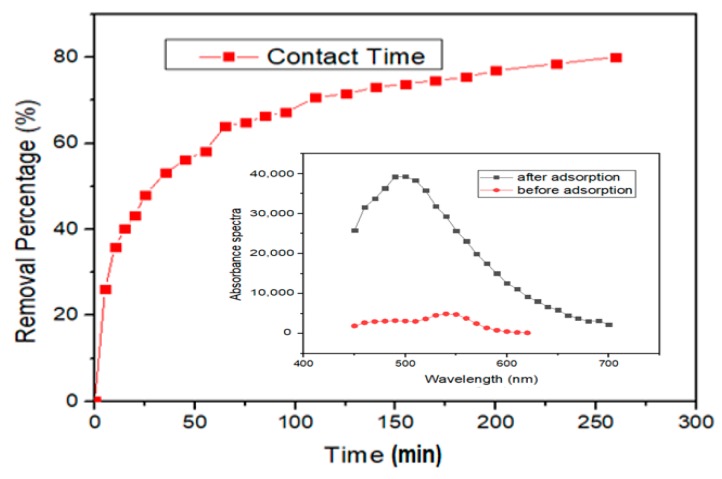
Effect of contact time on the removal efficiency of lead ions by CDs.

Several investigations have been reported regarding the quenching and detection effects that nanomaterials have on heavy metals. These properties of nanomaterials are due to their adsorptive capabilities [65,66]. In this study, batch adsorption was carried out to determine the CDs ability for adsorption of lead ions. Initial experiments were conducted to determine the contact time needed to achieve equilibrium for lead ion adsorption on CDs. The contact time for optimum adsorption was at 260 min with removal efficiency of 80.6% (Figure 7). The adsorption efficiency of CDs to remove lead ions at 80.6% have surpassed recent report by Xu et al. [67] who utilized iron hydroxyphosphate composites derived from waste lithium-ion batteries to remove 75% of lead ions in aqueous medium [67]. Mahar et al. [68] synthesized porous carbon nanofibers to adsorb lead ions in aqueous media and also reported a lower maximum adsorption efficiency of 79% [68]. In another study using *Enteromorpha* derived biochar, an efficiency of 54% was obtained for adsorbing lead ions [69]. Hence, the results provide clear evidence of CDs superiority for removal of lead ions in aqueous medium.

**Figure 8:**
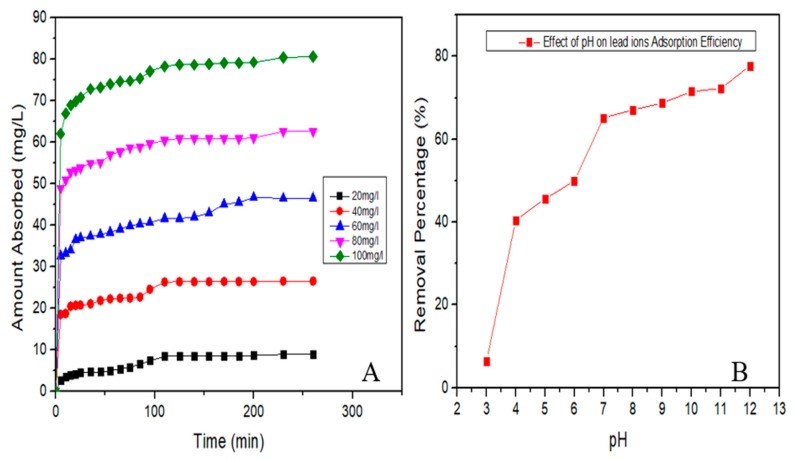
(**A**) initial concentration of lead ions and removal efficiency of CDs and (**B**) relationship between lead removal and pH.

Initial concentration of lead ion was analyzed to determine the effective adsorption attributes of CDs. The removal efficiency of CDs was high when the initial concentration of lead was at 100 mg/L (Figure 8A,B).

Figure 8A shows the validations of initial concentrations of lead on the adsorption efficiency of CDs. The removal efficiency of lead is proportional to the increase in initial concentration of lead (20–100 mg/L), due to the greater driving force existing at bulk concentration gradient [47].

Figure 8B shows the influence of pH on the adsorptive efficiency of carbon dots. A pH from seven to 13 was confirmed to be the best with efficiencies between 50–80% removal. This is because, at pH 7, the CDs exhibits a zeta potential of −31.7 mV which suggests existence of negative charge on the surface of CDs adsorbent. Zeta potential of CDs is the potential at the slipping plane that separates the mobile fluid from the fluid that remains attached to the solute. Thus, it can easily adsorb the positively charge lead ions [54,55].

#### 3.3.1. Adsorption Equilibrium

**Figure 9:**
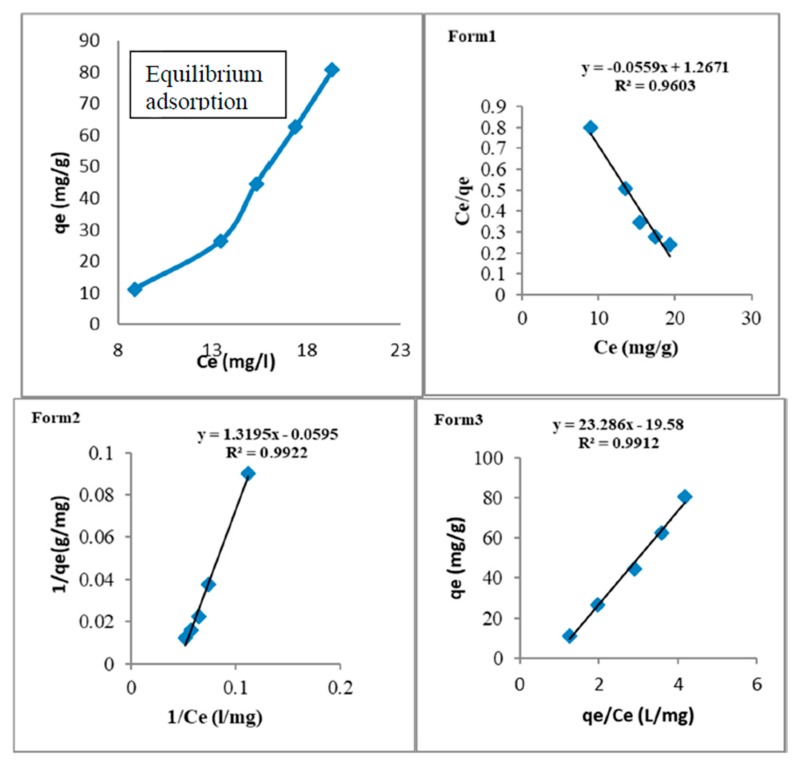
Equilibrium adsorption and the three forms of Langmuir isotherm for lead adsorption into CDs.

Adsorption equilibrium of lead adsorption by CDs was obtained with Langmuir forms one, two, and three by applying Equations (4)–(6). The R^2^ values were 0.9603, 0.9922, and 0.912 for Langmuir forms one, two, and three, respectively. The Langmuir isotherm model is a linear plot of the total amount of lead adsorbed (qe) against the equilibrium concentration (Ce) for CDs as shown in Figure 9 (Equilibrium of Adsorption). The equilibrium adsorption of lead increased with the concentrations of lead ions. Thus, suggesting a strong affinity of the lead ions for the functional surface sites of CDs [18,59].

The values of the Langmuir constant K_L_ and the monolayer capacity q_max_ can be evaluated from the three forms of Langmuir adsorption models. Comparing the three Langmuir forms in Figure 9, the Langmuir forms two and three have correlation coefficients R^2^ at 0.9922 and 0.9912, respectively compared to a lower R^2^ value obtained at Langmuir form one (0.9603). The coefficient of determination represents the variance about the mean that is used to analyze the fitting degrees of isotherms and kinetic models with experimental data. The Langmuir forms two and three are best suited for the study. Langmuir forms can be correlated with the variation of the suitable area and porosity of the adsorbent. Availability of CDs at nano dimension level offers vast surface area and pore volume availability for pore diffusion-sorption activities [50] that results in a higher adsorption capacity [18].

**Figure 10:**
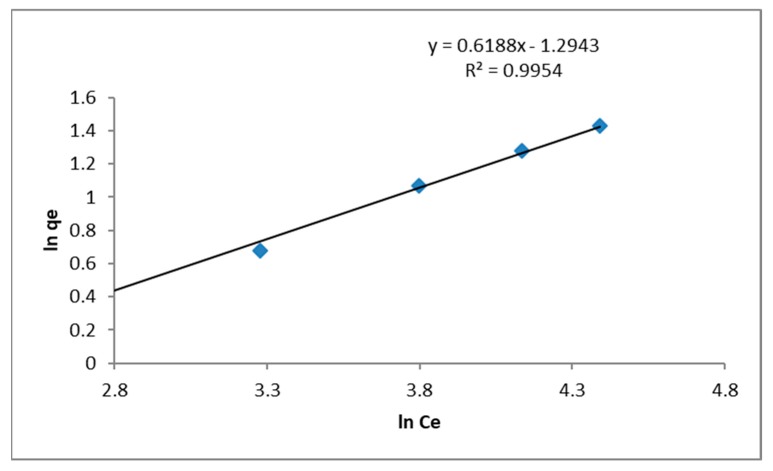
Freundlich Isotherm for CDs.

Based on Freundlich adsorption model, ln q_e_ against ln C_e_ was plotted as shown in Figure 10. The graph is a straight line that yields values of n (1.61) from the slope and K_F_ (0.0507) from the intercept respectively with coefficient of correlation of R^2^ = 0.9954. The slope 1/n ranges from zero to one, is a degree of adsorption intensity and surface uniformity. As the value approaches zero, the surface becomes more uniform [36]. The value of 1/n is 0.6188 and it indicates the heterogeneity of CDs. The value of exponent n in this investigation is greater than one which means the adsorption of lead on CDs is favorable.

The Freundlich isotherm confirms CDs as having defined surface heterogeneity and the exponential distribution of active sites [70]. The adsorption process as explored by both Langmuir and Freundlich isotherms have proven favorability of the adsorption process. This means CDs is a versatile substance with existence of heterogeneous surface areas suitable for effective monolayer and multilayer stacking conformed to both adsorption process.

#### 3.3.2. Adsorption Kinetics

The Lagergren equation was used in the determination of rate constant for adsorption of lead. The adsorption experiment was carried out at concentrations of 20–100 mg/L of lead, adsorbent dose of 0.1 g (the dosage of tapioca for synthesizing CDs) and contact time between 5 to 260 min. A graph of t/q_t_ against t was plotted in order to investigate this model.

##### Pseudo First and Second-Order Kinetic Models

**Figure 11:**
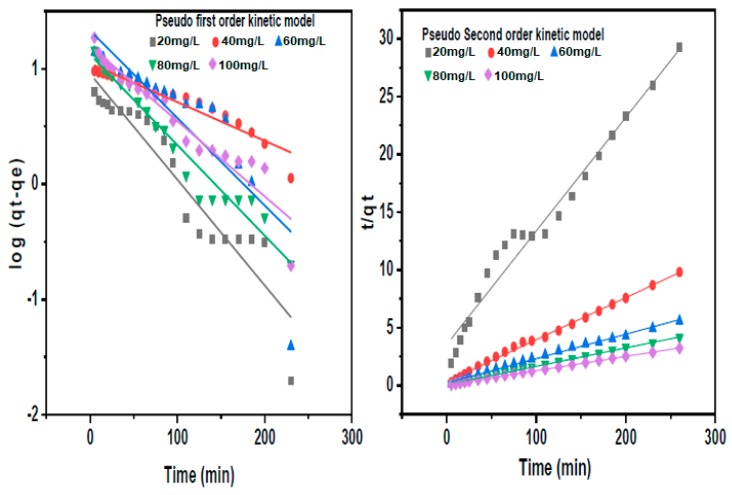
Initial Concentration of lead ions for first and second order kinetics.

Figure 11 shows linearized form of first order and second order kinetics model of lead concentration on CDs. The adsorption phenomenon of lead by CDs is best described by the second order kinetic model. The coefficient R^2^ for second order kinetic model at an initial concentration of 20 mg/L and 100 mg/L are 0.9668 and 0.9996, respectively. The calculated and experimental values for the adsorption of lead ions obeyed pseudo-second-order kinetic model [47]. Figure 11 shows the linear forms models for first and second order kinetics at varying lead concentration.

Pseudo-second-order rate constant (k_2_), q_e_, and q_cal_ were obtained from the slope and intercept of the graphs in Figure 11. From the calculated data (q_ecal_) values of pseudo-second-order kinetic model, there is compatibility trend when compared to the experimental data (q_exp_). The pseudo-second-order kinetic equation for adsorption is much similar to the universal rate law for chemical reaction [47]. Since the processes followed the pseudo second-order equation, it literally suggests that the adsorption is mainly by simple chemisorption reaction occurring between the lead ions and the surface functional groups of CDs [49,50].

## 4. Conclusions

The synthesized CDs is low cost without the need for tedious purification (environmentally friendly process). The CDs demonstrates to be a suitable adsorbent with remarkable properties which can have wide applications. Characterization techniques such as energy-dispersive X-ray spectroscopy (EDXs) and XPS have been utilized to determine quantitatively the compositional elements in CDs. The intensity counts and carbon content of CDs makes it suitable for adsorption studies of heavy metal (lead). CDs with zeta potential −31 mV means it is a stable hydrophilic substance. CDs capacity for absorption of heavy metal (lead) was confirmed with high efficiency of 80.6%. Both Langmuir and Freundlich indicates favorability of the adsorption process as shown by Langmuir Form two and three. Freundlich constant of 1/n indicates surface heterogeneity of CDs that is suitable for adsorption. The adsorption phenomenon of lead by CDs followed the second order kinetic model that is known to be similar to the universal rate law for chemical reaction. It literally suggests that the adsorption proceed mainly by simple chemisorption reaction occurring between the lead ions and the surface functional groups of CDs. The synthesized carbon dots are excellent fluorescent materials proposed for further applications in environmental fields of wastewater pollution detection, medical bio-imaging, and chemical sensing.

## Figures and Tables

**Table 1:** Atomic force microscopic statistics of CDs.

Parameter	Mean	Minimum	Maximum	Sigma
Total Count	32.000	32.000	32.000	0.000
Height	2.440 (nm)	0.409 (nm)	8.168 (nm)	1.875 (nm)
Area	1801.610 (nm^2^)	95.367 (nm^2^)	31,333.924 (nm^2^)	5432.314 (nm^2^)
Diameter	32.387 (nm)	11.019 (nm)	199.739 (nm)	35.284 (nm)
